# Antioxidant and antimicrobial effects of essential oils from two salvia species with in vitro and in silico analysis targeting 1AJ6 and 1R4U proteins

**DOI:** 10.1038/s41598-023-41178-2

**Published:** 2023-08-28

**Authors:** Souad Maache, Latifa Zbadi, Asmae El Ghouizi, Najoua Soulo, Hamza Saghrouchni, Farhan Siddique, Baye Sitotaw, Ahmad Mohammad Salamatullah, Hiba-Allah Nafidi, Mohammed Bourhia, Badiaa Lyoussi, Ilham Elarabi

**Affiliations:** 1 Laboratory of Natural Substances, Pharmacology, Environment, Modeling, Health and Quality of Life (SNAMOPEQ), Faculty of Sciences Dhar El Mehraz, USMBA, Fez, Morocco; 2 Public Health Laboratories at the Prefectural Delegation of Tangier Assilah, Tangier, Morocco; 3 Department of Biotechnology, Institute of Natural and Applied Sciences, Çukurova University, 01250 Balcalı, Adana, Turkey; 4 Department of Pharmaceutical Chemistry, Faculty of Pharmacy, Bahauddin Zakariya University, Multan, 60800 Pakistan; 5 Department of Biology, Bahir Dar University, P.O. Box 79, Bahir Dar, Ethiopia; 6 Department of Food Science and Nutrition, College of Food and Agricultural Sciences, King Saud University, 11, P.O. Box 2460, 11451 Riyadh, Saudi Arabia; 7 Department of Food Science, Faculty of Agricultural and Food Sciences, Laval University, 2325, Quebec City, QC G1V 0A6 Canada; 8 Laboratory of Chemistry and Biochemistry, Faculty of Medicine and Pharmacy, Ibn Zohr University, 70000 Laayoune, Morocco

**Keywords:** Biochemistry, Biotechnology, Drug discovery

## Abstract

The Middle Atlas is a Moroccan territory that serves as an abode to plants with incredible biodiversity, of which aromatic and medicinal plants that have been of folkloric use are a significant component. However, their effective utilization in modern medicine requires the characterization of their phytochemicals to facilitate their entry into drug discovery pipelines. Hence, this study aimed to characterize and investigate the antioxidant activity and antimicrobial effects of the essential oils (EOs) of *Salvia lavandulifolia* subsp. *mesatlantica* and *Salvia officinalis* L. by use of in vitro and in silico assays. Ten phytochemicals were identified in the EOs of *S. lavandulifolia*, while twenty phytochemicals were identified in *S. officinalis*. Camphor was the most abundant compound in both species, comprising 26.70% and 39.24% of the EOs of *S. lavandulifolia* and *S. officinalis*, respectively. The EOs of both plants exhibited significant DPPH free radical scavenging activity, with *S. lavandulifolia* and *S. officinalis* showing estimated scavenging rates of 92.97% and 75.20%, respectively. In terms of Ferric Reducing Antioxidant Power (FRAP), *S. officinalis* demonstrated a higher value (72.08%) compared to that of *S. lavandulifolia* (64.61%). Evaluation of the antimicrobial effects of the EOs of *S. officinalis* and *S. lavandulifolia* against microorganisms revealed bactericidal activities against *Proteus mirabilis* and *Bacillus subtilis* at low concentrations. It showed bactericidal activities against *Staphylococcus aureus* and *Candida albicans* at a relatively higher concentration. Molecular docking of antioxidant and antimicrobial proteins offers significant insights into ligand–protein interactions, facilitating the development of innovative therapeutics from the current study. Ultimately, this study identified the phytochemical composition of *S. lavandulifolia* and *S. officinalis* and highlighted their potential for therapeutic discovery.

## Introduction

Since prehistoric eras, therapeutic and aromatic plants have been used to enhance food’s flavor, color, scent, and pungency as well as to cure conditions like anticholinesterase activity, depression, and epilepsy^1,2^. The variety and abundance of bioactive chemicals obtained from organic, aqueous, and essential oil extracts control these biological and pharmacological activities^3,4^.

The most important medicinal and aromatic plants are listed in the *Lamiaceae* family, which is widely distributed worldwide^5,6^. The most famous examples are rosemary, oregano, basil, thyme, mint, lavender, and sage, which are widely used as medicinal and culinary herbs^7,8^. They have been used by ancient populations in old-style medicine and by present societies in modern medicine^9^. For example, 14 plant species of *Lamiaceae* have been demonstrated to have 95 medicinal usages and serve to remedy 13 dissimilar pathological collections in eastern Morocco and eastern Andalucía^10^.

One of the central genera of the *Lamiaceae* family is the *Salvia*. This genus is extensively used by both ancient and present populations in medicine, conservation of food, and esthetical purposes^11,12^. Equally, tea prepared from *Salvia* species has been defined by Grieve (in 1980) as a “highly serviceable stimulant tonic in debility of the nervous system”^13–15^. Also, *Salvia* species would relieve the symptoms of premenstrual syndrome and have an estrogenic effect as a hormonal regulator working on the female urogenital area^16^. Among *Salvia* species, *Salvia lavandulifolia* is the most commonly investigated herb in terms of pharmacology, ethnobotany, and biochemical composition^13,17,18^. As a spasmolytic, analgesic, antiseptic (with virucidal, bactericidal, and fungicidal actions), anti-inflammatory, sedative, anesthetic remedy, estrogenic, and anticholinesterase, *Salvia* species have been employed in traditional medicine^19^.

In Morocco, *S. lavandulifolia* is classified as one of the most utilized plants by the local population for managing numerous diseases^20^. It is distributed in Mountainous regions counting Atlas and Rif chains. It is recorded in the Watershed of Bigoudine located in Western High Atlas^21^, Tizi n’ Test area located in Taroudant province^22^, and Oulad Ali^20^. This herb can be encountered at elevations between 1 and 2000 m above sea level, and the plant rises mainly on calcareous and basic soils^21^. Local populations regularly use this aromatic plant as a folk cure to treat several ailments, and numerous of these pharmaceutical properties have been demonstrated in controlled laboratory investigations^23,24^. However, the laboratory studies showed a significant variability of chemical constituents depending on climate conditions, soil, and geographical location of the sampled plant, which is suggested to influence their therapeutic properties^17,25^.

Moreover, the laboratory and field investigations were limited to the species level. No studies have concerned the subspecies levels of *S. lavandulifolia* in Morocco, and this is suggested to add more valuable knowledge to medicinal plants in this country and the entire Northwest Africa. Equally, investigating such subspecies is recommended to discover new chemical constituents that could be used in modern pharmaceutical industries, food, and cosmetics. Another plant used in medicine in Morocco is *Salvia officinalis* L.^26^. It is utilized as an outdated herbal remedy against a diversity of diseases.

Further, *S. officinalis* is stated to have a wide array of biological properties, such as antibacterial, antioxidative properties, hypoglycaemic, fungistatic, anti-inflammatory, virustatic, eupeptic, astringent, and anti-hydrotic effects^26^. Equally, the essential oils of this species are used against a wide range of microorganisms, counting bacteria, fungi, and viruses^27,28^. However, some microorganisms, such as *Candida albicans* and *Pseudomonas aeruginosa* were resistant to the essential oils of *S. officinalis*^28^. However, the richness of medicinal plants with a wide range of chemical compounds, such as flavonoids and polyphenols, contributed to other biological activities^29,30^. For example, Nieto^31^ mentioned that the essential oils from three *Lamiaceae* plants counting sage, thyme, and rosemary, are promising to preserve food owing to their antimicrobial and antioxidant properties.

This study aimed to explore the biochemical constituents of essential oils extracted from wild *S. lavandulifolia* subsp*. mesatlantica* and *S. officinalis* L. Equally, their antioxidant capacity (DPPH, FRAP, and TAC) was evaluated, and their antimicrobial activity was tested against selected microorganisms by use of in vitro and in silico assays.

## Materials and methods

### Plant material

*Salvia lavandulifolia* subsp. *mesatlantica* and *S. officinalis* L. were retrieved in June 2021 from Immouzer Kandar, situated at 1359 m above sea level in Morocco’s Middle Atlas^32^. Notably, No approval is needed to collect *S. lavandulifolia* subsp. *mesatlantica* and *S. officinalis* L. in Morocco for research purposes.

Following the retrieval of the plants, they were raised under identical circumstances at Immouzer, a Mediterranean climate characterized by cold and damp winters and hot and dry summer. Notably, the average annual temperature of the area is 13.5 °C, and it experiences approximately 651 mm of rainfall on average per year. Dr M. Fanane at the botany division of the scientific institute of Rabat identified the plants using the Moroccan flora^33^, and specimens were placed in the herbarium of the scientific institute under the reference number RAB 112,040 for *S. lavandulifolia subsp. mesatlantica* and RAB 111,174 for *S. officinalis* L. Noteworthy, The leaves were dried under shade at room temperature for 15 days, then finely powdered and kept at 4 °C until further use.

### Extraction

Briefly, 100 g of each plant was separately subjected to hydrodistillation using a Clevenger-type apparatus for 3 h^34^. The EOs were stored at 4 °C until further analysis. The yield (w/w) was expressed in percent according to the following formula: Yield (%) = WEO/WP × 100, where WEO is the weight of essential oil and WP is the weight of dry plant extracted.

### Gas chromatography (GC/MS) analysis

By employing a Varian capillary column (CP-Sil 5CB, 50 m long, 0.32 mm in diameter, and 1.25 m in film thickness), GC–MS (Trace GC ULTRA, Thermo Fischer, France) was used to analyze the chemical composition of the oil extracted from *S. lavandulifolia* subsp. *mesatlantica* and *S. officinalis* L. The column temperature was scheduled to rise by 5 °C/min from 40 to 280 °C. One of the detectors (FID) had a set temperature of 260 °C, whereas the injector had a fixed temperature of 250 °C. The gas vector’s debit was set to 1 mL/min for nitrogen. The injected specimens contained 0.5 µL of diluted oil in a 10% hexane solution. Area peaks were used to calculate the proportions of each component in the oil^35^.

### Docking methodology

This work used molecular docking to evaluate the binding relationships between antioxidant proteins PDB ID: 1AJ6 and antimicrobial protein 1R4U with diverse ligands found in plant extracts, as shown in Table 1. Chem Draw Ultra^36^ produced the ligands, and Chem3D Pro^37^ optimized their shape via energy minimization. RCSB protein data bank (www.rcsb.org) provided the 3D crystal structures of antioxidant PDB ID: 1AJ6 and antimicrobial protein PDB ID: 1R4U. Preprocessing removed water molecules and non-binding heteroatoms from protein structures. Proteins were protonated by adding hydrogen atoms and partial charges. Grid boxes were created around the active sites of proteins 1AJ6 and 1R4U utilizing the ligand-binding residues as a reference to search for ligand binding during docking simulations^38^. Autodock tools saved optimized ligands and protein structures in pdbqt format^39,40^. Autodock Vina, a suitable algorithm, conducted molecular docking. Within grid boxes, ligands were flexible docked onto protein active sites. Docking simulations examined ligand conformations and orientations to determine binding modes^41^. The docking programme scored ligand–protein complexes based on binding energy. Lower binding energy values suggested greater ligand–protein interactions. To find the best binding interactions, docked complexes were rated by binding energy^40,41^.

**Table 1:** Recorded chemical constituents in the essential oils (EOs) of *S. officinalis* L*.* and *S. lavandulifolia* subsp. *mesatlantica*.

Chemical compounds	%	
	*Salvia officinalis* L.	*Salvia lavandulifolia* subsp. *mesatlantica*
Camphor	26.7	39.24
β Thujone	17.14	0
Eucalyptol	16.96	22.01
α Pinene	7.01	5.55
Ledol	6.83	2.63
Thujone	5.08	0
Camphene	4.18	9.71
Caryophyllene	2.17	0
Epimanool	1.7	0
α Humulene	1.64	0
Borneol	1.64	6.75
Humulene epoxide2	1.49	1.86
Caryophyllene oxide	1.33	2.56
D-Limonene	1.31	2.93
β Pinene	1.03	6.76
β Myrcene	0.85	0
Fenchyl acetate	0.83	0
1-Terpinenol	0.75	0
Isoaromadendrene epoxide	0.69	0
Humulenol-II	0.68	0

The docked complexes with the best binding energies were analyzed to determine the ligand–protein interactions. BIOVIA Discovery studio visualized key binding residues and interactions such as hydrogen bonds, electrostatic interactions, and hydrophobic interactions^41^.

### Determination of antioxidant activity

#### Scavenging of the free radical DPPH

The antioxidant potentials of the EOs in both *Salvia* species were evaluated based on their ability to scavenge 2,2-diphenyl-1-picrylhydrazyl (DPPH) free radicals. The techniques outlined by Tepe et al., Farahpoul et al., and DiCiaulaa et al*.*^42–44^, were utilized with slight modifications (e.g., DPPH was prepared at 2.5% in ethanol). 750 µL of the DPPH solution was mixed with 50 µL of each plant sample at different concentrations (tenfold serial). The resulting mixture was vortexed for 15 s and then placed at room temperature for 1 h. The absorbance of the mixture was recorded against a blank at 517 nm using a spectrophotometer. Ascorbic acid, prepared under similar circumstances, was utilized as the reference antioxidant. The results were expressed in percentage reduction of DPPH using the equation below:

1 $${[(\mathrm{Abs}}_{\mathrm{control}}-{\mathrm{Abs}}_{\mathrm{sample}} / {\mathrm{Abs}}_{\mathrm{control}}) \times 100]$$

Abs_control_ is the recorded absorbance of the control reaction (comprising all components except EOs) and Abs_sample_ is the absorbance recorded for essential oils tests. The linear regression equation was employed to graphically calculate the essential oil’s concentration that inhibits fifty percent of the original concentration (IC_50_) of DPPH;

#### Total antioxidant capacity (TAC)

The total antioxidant capability (TAC) of the essential oils was calculated based on the method reported by Farahpour et al.^43^. Notably, the ammonium molybdate component was prepared by mixing 0.6 M sulfuric acid and sodium phosphate (28 mM) solution with ammonium molybdate (45 mM). 1 mL from this mixture was added to 50 µL of EOs prediluted with tenfold serial using ethanol. The resulting mixture was coated and subjected to a temperature of 95 °C for 90 min in a heat block. Subsequently, the absorbance of the resulting mixture was measured with a UV–visible spectrophotometer at 695 nm and then compared to that of the control. The obtained results were related to a reference antioxidant, namely ascorbic acid. Ascorbic acid was utilized as the regular calibration, and the findings were expressed in mg of eq of ascorbic acid per g of dry matter.

#### FRAP

The reducing power (FRAP) of the essential oils was estimated using the iron reduction scheme described by Oyaizu^45^. For this study, 50 µL of the sample tenfold serially diluted was mixed and was mixed with 250 µL of sodium phosphate (0.2 M) buffer (with a pH equal to 6.6) and 250 µL of potassium ferricyanide (1%). Subsequently, the resulting mixture was capped and incubated in the dark at 50 °C for 20 min. Furthermore, 10% of trichloroacetic acid (250 µL) was added. After centrifugation (speed & time), 250 µL of the supernatant was recuperated and mixed with 250 µL of distilled water and 0.1% of ferric chloride (60 µL). Finally, the absorbance was measured at 700 nm, using ascorbic acid as a standard.

### Antimicrobial activity

#### Inoculum preparation

The essential oils extracted from both plants were tested against a series of pathogenic microorganisms sequestered and identified in the bacteriology laboratory of Hassan II Hospital in Fez. The microorganisms include Gram-positive bacteria, namely *Bacillus subtilis* DSM 6333 and *Staphylococcus aureus* ATCC 6633, Gram-negative bacteria, namely *Proteus mirabilis* ATCC 29,906, and a fungal strain, namely *C. albicans* ATCC 10,231. The bacteria strains were inoculated by streaking on Mueller Hinton agar, while the yeast was inoculated in Sabouraud dextrose agar. Furthermore, the bacteria were incubated for 18–24 h at 37 °C, while yeast was incubated at 30 °C for the same period. Three well-isolated colonies are picked and emulsified in 10 mL of sterile 0.9% phosphate-buffered physiological water using a vortex. Dilutions were made to standardize the bacterial suspension and adjust it to 0.5 McFarland.

#### Disc method for EOs

The antimicrobial effect of essential oils (Eos) was determined by the disk diffusion method as described in^46,47^ for bacteria and fungi, respectively. The diffusion was performed on 6 mm diameter Wattman paper discs, sterilized, and impregnated at a rate of 10 µL/disc and deposited on the surface of an agar medium previously spread with a microbial suspension of the tested microorganism at a rate of 100 µL/5mL of Mueller Hinton Agar (MHA) (The agar medium at a temperature of 45 °C).

Two controls were performed on a negative control with 10 µL of sterile distilled water in the presence of 2% DMSO and an antibiotic disc as a positive control. The dishes were left for 15–30 min at room temperature and then incubated at specific growth conditions of bacteria (37 °C for 18–24 h) and yeast (30 °C for 48 h). Inhibition zones (IZ) around disks were measured and recorded in mm. Noteworthy, the diameters of the inhibition zones were assessed in mm, with triplicate for each test. Ultimately, the values obtained were expressed as means ± SD of triplicates.

#### Determination of minimum inhibitory concentration (MIC) and minimum bactericidal concentration (MBC)

The MIC is the lowest dilution at which no microbial growth is observed. To determine the MIC values of the essential oils, 50 μL of sterilized nutrient broth or Brain heart infusion (BHI) was distributed into each well of a 96-well microplate with an Inoculum (50 μL) of the microorganism of interest along with tenfold serially diluted essential oils. The plates were incubated for 24 h at 37 °C or 30 °C and then carefully examined using 0.015% Rizasurine to check the presence of microorganisms. The MBC and MFC denote the minimum concentration that eliminates the microorganisms; these indexes were determined from the MIC test, corresponding to the concentration of EOs that inhibit bacterial/fungal growth. It is known that the MBC/MIC ratio ≤ 2 is considered bactericidal, and the ratio > 2 is considered bacteriostatic (inhibition).

### Statistics

Three independent measurements were made for each tested parameter (DPPH, TAC, and FRAP). The results were presented as means ± SD, and all studied parameters were tested for normality and homogeneity of variance. Furthermore, TAC and FRAP were compared in essential oils of the *Salvia* species being analysed using a T-test (two groups). To test for the correlation between microorganisms (n = 4) and inhibitory effects (IZ, MIC, and MBC) of the OEs (n = 2) and chemical antibiotics (n = 2), Principal Component Analysis (PCA) was utilized, and all tests were performed using the STATGRAPHICS centurion XII software. For the statistical significance, the *p*-value taken was 0.05.

### Plant collection approval

No approval is needed to collect *S. lavandulifolia* subsp. *mesatlantica* and *S. officinalis* L. in Morocco for research purposes.

### IUCN policy statement

The collection of plant material complies with relevant institutional, national, and international guidelines and legislation.

## Results and discussion

### Chemical composition and yield of EOs

Using the extraction method, the yield of *S. officinalis* L. and *S. lavandulifolia* subsp. *mesatlantica* EOs were 1.37 ± 0.8% and 0.86 ± 0.3% respectively. Similarly, the concentration of EOs in both plants was variable. In *S. officinalis*, the concentration was 750 mg/mL compared to 600 mg/mL in EOs of *S. lavandulifolia*. For *S. officinalis*, similar results were recorded currently in areal parts. Hazrati et al.^46^ investigated the yield of EOs based on microwave gravity and hydrodiffusion (MHG), in addition to microwave-generated hydrodistillation (MGH) for extraction. Results showed that harvesting from 4:00 to 6:00 p.m. revealed the maximum EO percentage estimated at 1.14%, whereas harvesting from 04:00 to 06:00 a.m. indicated the smallest EO percentage estimated at 0.599%.

In another study, the yield in essential oils from leaves *S. officinalis* was 2.25% for essential oil dried for twenty-one days and about 1.91% during seven days^28^, which are variable compared with our results. Therefore, the variation of yield and concentration of essential oils of *S. officinalis* between our samples and those of previous studies is suggested to be related to the difference in used extraction methods, parts of the plants used and drying techniques. Boutebouhart et al.^28^ investigated the effect of extraction and drying methods on the yield of essential oils from *S. officinalis*. The yield was significantly variable depending on the extraction method, drying technique, and period. On the other hand, despite the lower yield of *S. lavandulifolia* subsp. *mesatlantica*, this is the first study that focuses on the extraction of EOs from this wild subspecies. Therefore, detailed studies are needed to get deep insight into the effect of extraction methods, solvents, and parts of the plant on the yield and concentrations.

**Figure 1:**
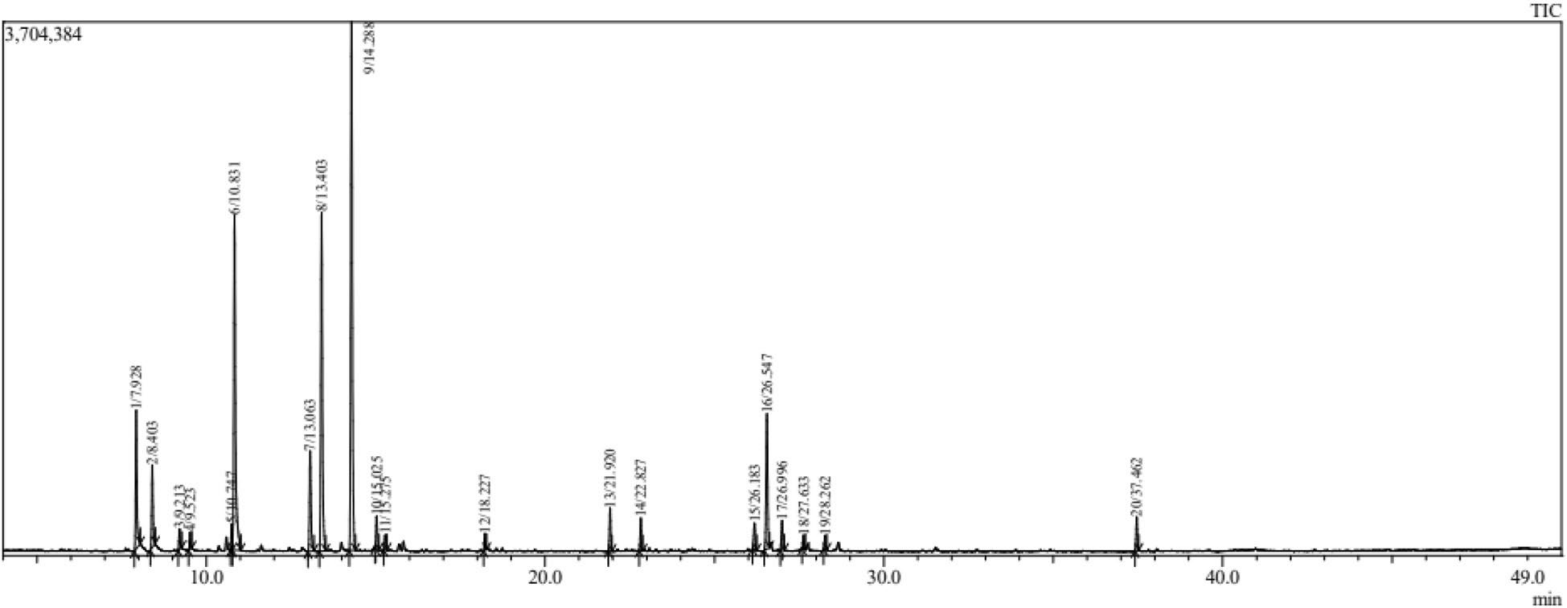
Chromatogram of essential oil from *Salvia officinalis* L.

**Figure 2:**
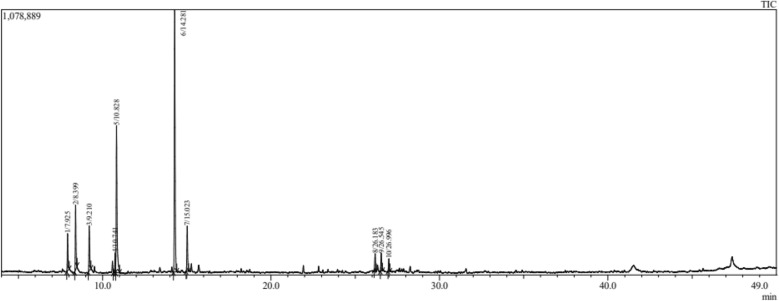
Chromatogram of essential oil from *Salvia lavandulifolia.*

The important result for the composition (The chemical profile of EOs in both *Salvia* species) is presented in Table 1. The GC/MS analysis revealed 20 (total%) and 10 (total%) chemical constituents in EOs distilled from *S. officinalis* (Fig. 1) and *S. lavandulifolia* (Fig. 2), respectively. The main constituents in *S. officinalis* were Camphor (26.70%), β Thujone (17.14%), and Eucalyptol (16.96%). While, Camphor (39.24%), Eucalyptol (22.01%), and Camphene (9.71%) were the dominant ones in *S. lavandulifolia*. Both EOs share the dominance of Camphor and Eucalyptol and 10 common phytochemicals representing the total mixture identified in *S. lavandulifolia.*

This study recorded 20 chemical constituents in EOs from *S. officinalis* L., compared to only 10 constituents in *S. lavandulifolia* subsp. *mesatlantica*. In *S. officinalis*, Camphor (26.70%), followed by β Thujone (17.14%), and Eucalyptol (16.96%) were the major chemicals recorded in EOs. Hazrati et al.^46^ recorded 32 components in EOs extracted from areal parts of *S. officinalis*, and the major elements were cis-thujone (34.38–46.18%), followed by 1,8-cineol (8.70–11.07%), and camphor (9.65–14.38%). In another study, Al-Mijalli et al.^48^ recorded 14 compounds in essential oils of *S. officinalis* collected from wild areas of Morocco. Thujone (33.77%), followed by Caryophyllene (12.28%), Humulene (12.19%), and Camphor (11.52%) were the primary elements. The lower number of constituents could be due to extraction method, climate conditions, and or soils characteristic in sampled sites.

Maric et al.^49^ investigated the effect of locality altitudes and development stages on the volatile chemicals of *S. officinalis* L. from Bosnia and Herzegovina. They revealed that both factors impacted the diversity of chemicals. The quality of chemical composition was constant, while the quantity was variable depending on the altitude and development stage of *S. officinalis* L. In other studies, Boutebouhart et al.^28^ and Saša Đurović et al.^50^ demonstrated that the extraction method affects the chemical composition of essential oils from *S. officinalis* L.

On the other hand, the diversity of bioactive molecules in the essential oils of *S. officinalis* compared to *S. lavandulifolia* increases its biological properties since these compounds are responsible for the biological activities of essential oils and extracts in plants^47,51,52^. Equally, this is the first study to characterize the chemical profile of *S. lavandulifolia* in Morocco and the entire Mediterranean area. Compared with other subspecies of *S. lavandulifolia*, Zrira et al.^53^ recorded 34 chemical constituents in essential oils from *S. lavandulifolia* Vahl collected from Morocco. The major components were camphor (16–31%), followed by 1,8–cineole (13–19%), and β-pinene (8–13%). In another study, *AENOR*^54^ recorded 12 chemical compounds in the Iberian samples (leaves) of *S. lavandulifolia* Vahl. A study by Jordán et al.^55^ reported a wide range of chemicals in *S. lavandulifolia* subsp. *vellerea* from South-Eastern Spain. The major chemicals were 1,8-cineol-camphor (23.9–40.7%) and 1,8-cineol-camphor (36.9–31%). The variation of quality and quantity of chemicals in essential oils of our samples and those of the *S. lavandulifolia* Vahl is suggested to be governed by the synthetic pathways of secondary metabolism in both subspecies. However, more investigations are needed to clarify this issue between subspecies and species of the same genus.

### Antioxidant activity

#### Scavenging of the free radical DPPH

The effect of essential oils extracted from both *S. officinalis* and *S. lavandulifolia* on DPPH inhibition is summarized in Table 2. Essential oils of both medicinal plants showed important free radical (DPPH) scavenging action. However, the value of DPPH (free radical scavenging action) was significantly higher in *S. lavandulifolia* (92.97%) compared to *S. officinalis* (75.20%) (*p* < 0.001). On the other hand, the IC_50_ of *S. lavandulifolia* was estimated at 34.55 mg/mL, while the IC_50_ of *S. officinalis* was estimated at 40.72 mg/mL.

**Table 2:** The effect of essential oil of *S. officinalis* and *S. lavandulifolia* on DPPH and TAC.

Essential oils	Plants		T-test		
	*Salvia lavandulifolia*	*Salvia officinalis*	*t*	*df*	*P*-value
DPPH free radical scavenging (%)	92.97 ± 1.01	75.2 ± 2.31	12.194	4	0
IC_50_ (mg/mL)	34.558 ± 0.99	40.72 ± 0.98	− 8.902	4	0.001
Total Antioxidant Capacity (mg EAA/g DW)	49.941 ± 1.00	36.349 ± 0.99	16.722	4	0

#### Reducing power (FRAP)

**Figure 3:**
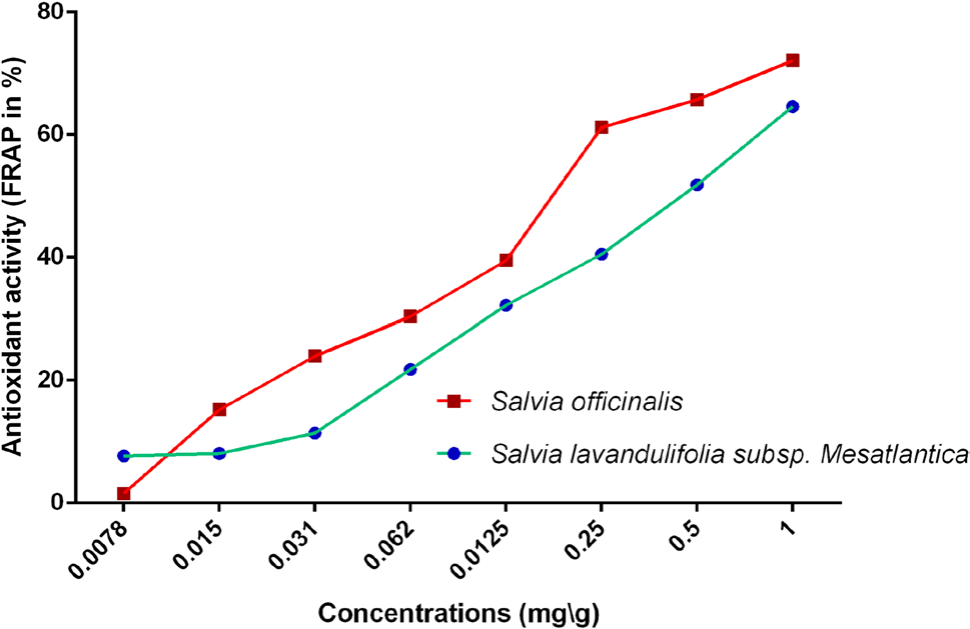
antioxidant activity (FRAP assay) of essential oils of *S. officinalis* and *S. lavandulifolia.*

The results of antioxidant activity, measured by the FRAP method, of EOs are presented in Fig. 3. Generally, *S. officinalis* exhibited higher potential than *S. lavandulifolia*. The FRAP activity for both species varied proportionally to the concentration, where the maximum inhibition values (72.08% ± 0.75 for *S. officinalis* and 64.61% ± 1.15 for *S. lavandulifolia*) have been recorded at 1 mg/mL. The statistical tests confirmed the significant difference of FRAP between essential oils of both plants (t = 9.658, df = 4, *p* = 0.001).

#### Total antioxidant capacity (TAC)

A comparison of total antioxidant capacity (TAC) between the essential oils of *S. officinalis* and *S. lavandulifolia* is presented in Table 2. Essential oils of both plants showed interesting antioxidant capacity. However, the highest total antioxidant capacity was recorded in *S. lavandulifolia* (49.941 mg EAA/g DW) compared to *S. officinalis* (36.349 mg EAA/g DW).

The antioxidant activity was heavily investigated for essential oils of *S. officinalis* compared to *S. lavandulifolia*^28,56–58^. Boutebouhart et al.^28^ investigated the antioxidant activity in essential oils from leaves of *S. officinalis* L. cultivated in Algeria. The essential oils (EOs) were obtained by different extraction techniques: microwave-assisted hydrodistillation (MAHD), conventional hydrodistillation (HD) technique, and steam distillation (SD). In Sage MAHD the Scavenging of the Free Radical DPPH ranged from 7.43 ± 1.06% to 34.72 ± 0.63%, 9.69 ± 2.12% to 36.75 ± 1.25% for Sage HD, and 9.75 ± 1.23% to 40.25 ± 0.85% for Sage SD^28^. Currently, Tundis et al.^47^, evaluated the antioxidant activity in three samples of *S. officinalis* essential oils from Italy: samples from Orsomarso (S1); from Civita (S2), and from Buonvicino (S3). The results showed that the IC_50_ of S1 was 31.58%, 35.33% in S2, and 32.52% in S3. These values are inferior compared to our results, and this difference is suggested to be controlled by the origin of used materials. In our case, we used wild *S. officinalis*, known for their higher antioxidant activities than cultivated varieties^48,59^. On the other hand, Asensio-S.-Manzanera et al.^60^, investigated the antioxidant activity in *S. lavandulifolia* and two other plants *Lavandula latifolia* and *Thymus mastichina* collected from Spain. The scavenging effect on DPPH ranged between 61 and 89% in spike lavender. These values are very close to our results in Moroccan wild *S. lavandulifolia.* The similarity of our results with those studies concerning the other subspecies of *S. lavandulifolia* could be due to similarity of extraction methods, used protocols, and the origin of samples (all samples were from the wild and Mountainous areas).

Tundis et al.^47^ evaluated the antioxidant activity in essential oils of *S. officinalis* from three samples in Italy: *S. officinalis* from Orsomarso (S1), *S. officinalis* from Civita (S2); *S. officinalis* from Buonvicino (S3). The obtained results showed that the FRAP of S1 was 3.11 ± 1.61 μM Fe (II)/g, 0.73 ± 0.09 μM Fe (II)/g found in S2, and 1.56 ± 1.02 μM Fe(II)/g found in S3. These authors confirmed that the antioxidant activity of the studied species varies depending on the sampled area, which suggests the potential effects of climate, soil, and other factors. Concerning the subspecies of *S. lavandulifolia* (subsp. *Mesatlantica*), our study presents the first investigation of its antioxidant activity, which is suggested to offer data for future studies.

### Antimicrobial activity

#### Determination of inhibition zone and minimum inhibitory concentration MIC and minimum bactericide concentration MBC

**Figure 4:**
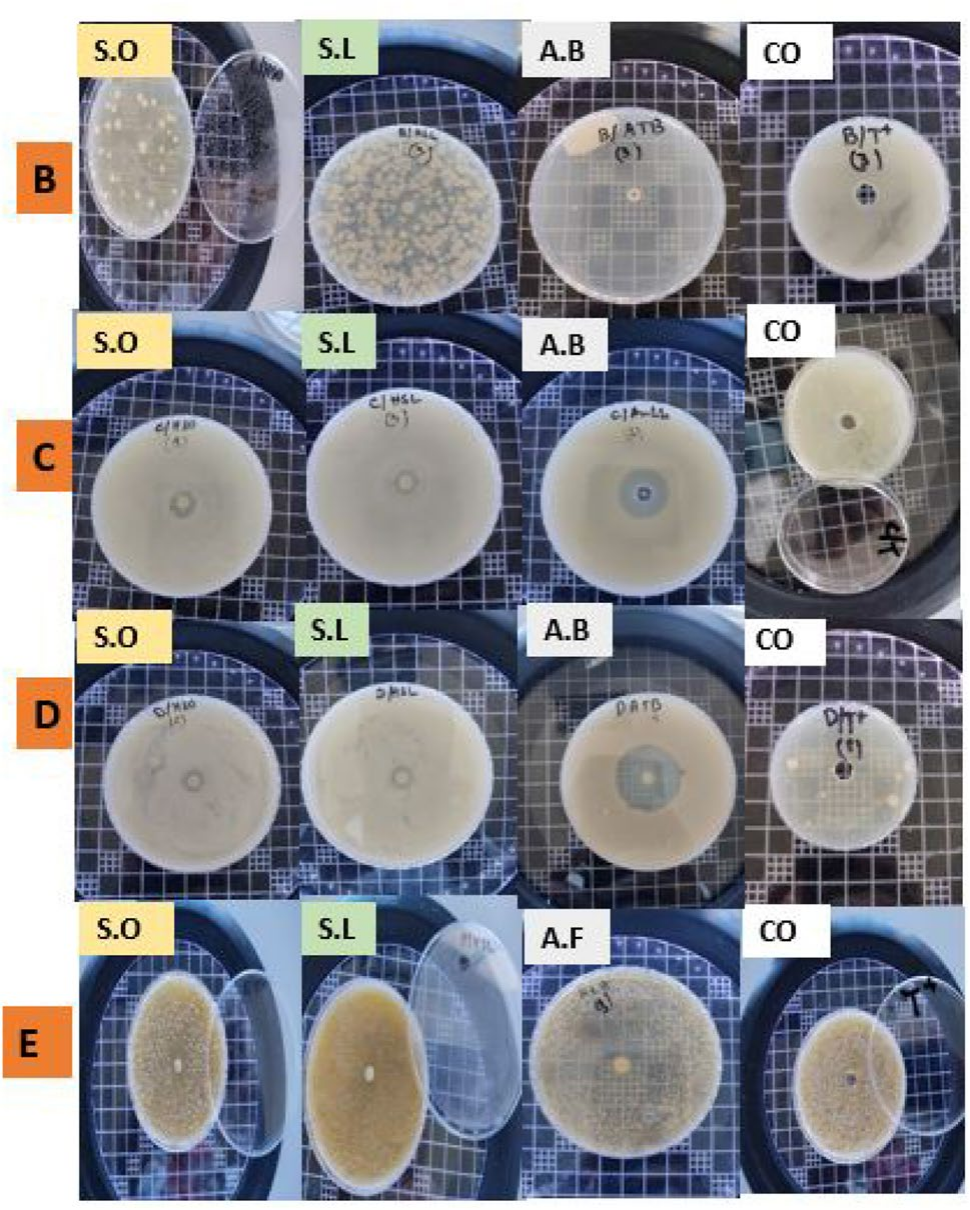
Bactericidal effect of SO and SL samples tested against bacteria and yeasts. (B = *Staphylococcus aureus*; D = *Proteus mirabilis*; C = *Bacillus subtilis*; E = * C. albicans*; SO = *S. officinalis*; SL = *S. lavandulifolia* subsp. *Mesatlantica*; A.B = antibiotic; A.F = antifungal; CO = control with bacteria only).

The results of the inhibitory effects of essential oils of both *S. officinalis* and *S. lavandulifolia* against tested microorganisms are presented in Table 3. Essential oils of both plants showed important and variable inhibitory effects against tested bacteria and yeast. The best antibacterial activity was shown by the essential oil from *S. officinalis* and the essential oil from *S. lavandulifolia,* with the smallest MIC values against *P. mirabilis* (0.29 mg/mL), as well as against *B. subtilis* and *P. mirabilis* (1.87 mg/mL each), respectively. Similarly, the best MBC values obtained from *S. officinalis* and *S. lavandulifolia* essential oils are against *P. mirabilis* and *B. subtilis.* *C. albicans*' MIC values ranged from 3.75 to 4.69 mg/mL. While *S. aureus* required an even higher concentration of the *S. officinalis* essential oil (18.75 mg/mL) to inhibit bacterial growth, it only needed 3.75 mg/mL from the *S. lavandulifolia* essential oil. Lower MBC values were found against *P. mirabilis* using *S. officinalis* essential oil (1.17 mg/mL), as well as against *P. mirabilis and B. subtilis* using *S. lavandulifolia* essential oil (3.75 mg/mL)*.* Based on the MIC/MBC ratio, essential oils of both *Salvia* species showed bactericidal effects against all tested microorganisms (Fig. 4) (MBC/MIC ratio ≤ 2 is considered as bactericidal and > 2 is considered as bacteriostatic (inhibition)).

**Table 3:** Comparison of inhibition effects (MIC, MBC, and MBC/MIC) between essential oils of *S. officinalis* and *S. lavandulifolia* against tested microorganisms.

Essential oils	*Parameters* (mg/mL)	*Staphylococcus aureus*	*Proteus mirabilis*	*Bacillus subtilis*	*Candida albicans*
*Salvia officinalis*	MIC	18.75	0.29	2.34	4.69
	MBC	37.5	1.17	4.69	9.37
	MBC∕MIC	2	4.03	2	1.99
*Salvia lavandulifolia*	MIC	3.75	1.87	1.87	3.75
	MBC	7.5	3.75	3.75	7.5
	MBC∕MIC	2	2	2	2

The antimicrobial effect of extracts prepared from diverse parts of *S. officinalis* was investigated heavily^28,61^. Boutebouhart et al.^28^ investigated the inhibitory effects of essential oils from areal parts (leaves) of cultivated *S. officinalis* L. against (i) Bacteria counting *S. aureus* (ATCC 6538P), *B. subtilis* (ATCC), *Escherichia coli* (ATCC 43,300), and *P. aeruginosa* (ATCC 27,853), (ii) Filamentous fungi counting *Aspergillus carbonarius* (M333) and *Umbelopsis ramanniana* (NRRL 1829), and (iii) Yeast namely *C. albicans* (ATCC 10,259). Except for *P. aeruginosa* (ATCC 27,853) and *C. albicans* (ATCC 10,259), essential oils of *S. officinalis* showed significant inhibitory effects with variable inhibition zones. Aćimović et al.^61^ tested the inhibitory effects of essential oils extracted from cultivated *S. officinalis* in Serbia against *S. aureus* (8684), *P. aeruginosa* (8762), *Enterobacter cloacae* (8923), *C. albicans* (8937), *E. coli* (8965), *Klebsiella oxytoca* (8929), and blood cultures *Klebsiella pneumoniae* (H2807) and *S. aureus* (H2846). In another study, essential oils of *S. officinalis* showed MIC values estimated at 191.83 against *E. coli*, 383.00 against *K. pneumonia*, > 512 against *E. faecalis*, and 96.05 against *S. aureus*^7^. All tested microorganisms were sensitive to *S. officinalis* essential oils. These findings confirm the large antimicrobial spectrum of *S. officinalis* essential oils against bacteria, fungi, and yeast. These biological activities are directly supported by the diversity and abundance of bioactive molecules in essential oils of *S. officinalis*^61,62^.

As far as our literature survey could ascertain, there is no published data on the antimicrobial activity of *S. lavandulifolia* subsp. *mesatlantica*. The inhibition rates (MIC and MBC) recorded for all tested bacteria and fungi are significantly inferior compared to those recorded for *S. officinalis*. In terms of comparison, previous studies^7,63^ investigated the composition of essential oils in four *Salvia* species including *S. lavandulifolia*, *S. sclarea, S. officinalis*, and *S. triloba*. The Minimum Inhibitory Concentration (MIC) technique and the disk diffusion method were used to investigate their antibacterial activity against 10 pathogens. Gram-positive microorganisms showed greater susceptibility to essential oils. Notably, 2.31 mg mL^−1^ was the MIC of *S. lavandulifolia* essential oils against *S. aureus*, while MIC againt *Shigella flexneri* was determined to be 9.25 mg mL^−1^. The major compounds behind this antimicrobial activity were α–β-thujone, camphor, and 1,8-cineole^7,63^. These elements are abundant in the essential oils of our samples.

**Figure 5:**
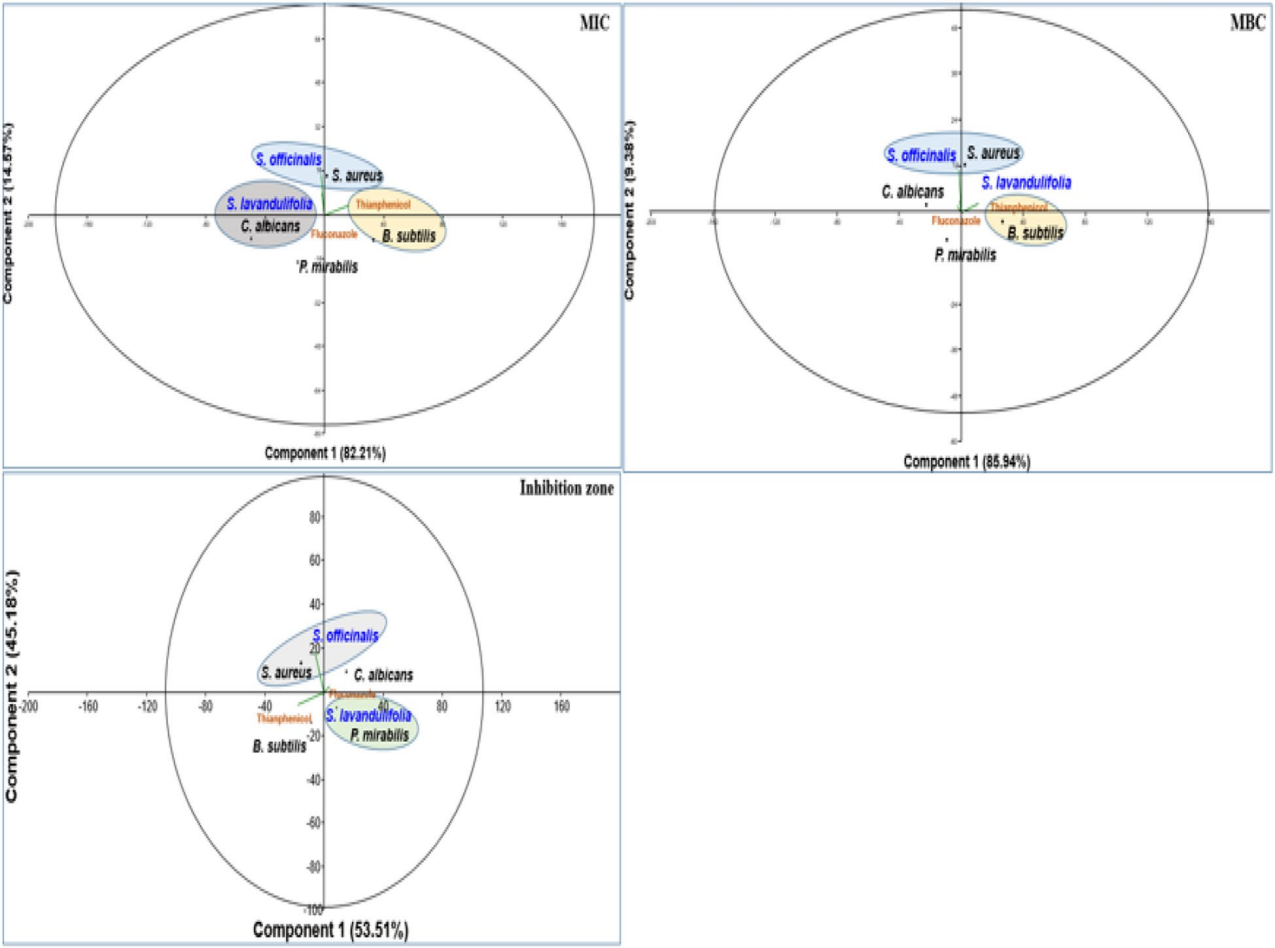
PCA plot showing the efficacy of tested essential oils of both plants (*Salvia officinalis* and *S. lavandulifolia*) and commercial antimicrobials.

The results of inhibition zones against tested microorganisms are presented in (Fig. 5, Table 4). The inhibition zones were variable depending on the used essential oils and tested microorganisms. In SA, the IZ were significantly superior in the essential oil of SO and TH compared to SL. In PM, the IZ was significantly superieur in TH, while it was similar in essential oils of both SL and SO. In BS, the IZ was significantly superior in essential oil of both SL and SO compared to TH. Against CA, the IZ was significantly superior in essential oils of SO, while it was similar between essential oil of SL and FL.

**Table 4:** Comparison of inhibition zone depending on using essential oils and antimicrobial substances (SO: *Salvia officinalis*; SL:* S. lavandulifolia;* TH*: Thianphénicol;* FL*: Fluconazol*) and tested microogranisms.

	*Staphylococcus aureus*	*Proteus mirabilis*	*Bacillus subtilis*	*Candida albicans*
SO	33.66 ± 5.68***a	12.33 ± 0.57*b	14 ± 1.73*a	25.33 ± 1.15**a
SL	18 ± 5.5*c	13.66 ± 1.52*b	13.66 ± 0.57*a	15.33 ± 4.16*b
TH	26.33 ± 0.57**b	43 ± 12.76***a	10.33 ± 1.52*b	–
FL	–	–	–	9.66 ± 2.88*b

*** > ** > * (* equivalent to *p* < 0.05) (comparison for each tested substance).

a > b > c (comparison for each microorganism).

In terms of comparison among the microorganisms for the same essential oils, IZ was also variable. For SO, the highest IZ was recorded in against ST, followed by CA, while it was similar between PM and BS. In contrast, the IZ of essential oil from the SL was similar among all microorganisms.

The PCA plot confirms the efficacy of each essential oil against tested microorganisms. Essential oils of SO are effective against SA, while essential oils of SL are effective against PM.

In North Africa, Hayouni et al.^64^ tested the inhibitory effect of essential oils from Tunisian *S. officinalis* L*.* against *Salmonella* inoculated in minced beef meat. The results showed that the inhibition zone varied from 9 to 36 mm, which agrees with our results. This is logical since our samples and those of Tinisia are from similar contexts, including climate (i.e. both from North Africa), used parts (leaves) and extraction methods. Similar results were also recorded in samples of SO from Agadir in the South of Morocco with an inhibition zone variable between 5 and 35 mm^65^, which are similar to our results for SO.

### Molecular docking

Table 5 presents the results of the molecular docking of various ligands with the antioxidant protein target 1AJ6. The table includes information on binding affinity, hydrogen-binding Interaction, hydrophobic Interaction, and electrostatic Interaction with distances in Angstrom for each ligand. The binding affinity, represented by the ΔG (kcal/mol) values, indicates the strength of the ligand and protein interaction. A lower ΔG value indicates a stronger binding affinity between the ligand and protein. Based on the table, the ligand “Iso-aromadendrene epoxide” has the strongest binding affinity with a ΔG value of − 6.4 kcal/mol. The table also includes information on the type of interactions that occur between the ligands and the protein target. Hydrogen bonding, hydrophobic, and electrostatic interactions are considered the most significant types of interactions in molecular docking studies.

**Table 5:** Molecular docking score, Hydrogen binding, hydrophobic and electrostatic interactions with distances in Angstrom for investigated ligands using protein target 1AJ6.

Ligands with 1AJ6	Binding Affinity, ΔG (kcal/mol)	Hydrogen-Binding interaction, Residue (Distance Å)	Hydrophobic Interaction, Residue (Distance Å)	Electrostatic interaction, Residue (Distance Å)
Camphor	− 4.4	–	ARG19 (4.34)	–
β-Thujone	− 5.1	ARG76 (3.05)	ILE78 (5.05)	–
		GLY77 (1.90)		
Eucalyptol	− 4.7	GLY119 (3.47)	ILE94 (5.45)	–
			ALA100 (3.90)	
α-Pinene	− 4.4	–	ILE94 (4.68)	–
			ALA100 (4.26)	
			ALA100 (4.15)	
Ledol	− 5.6	–	PHE41 (5.02)	–
			ARG190 (4.35)	
Thujone	− 5.1	–	ILE78 (5.38)	–
Camphene	− 4.2	–	ILE94 (5.29)	–
			ALA100 (3.89)	
Caryophyllene	− 5.7	–	PHE41 ((4.94)	–
13-Epimanool	− 6.1	ASP45 (2.18)	PHE41(4.85)	–
		ARG190 (2.98) ARG190 (2.169)	PHE41 (5.38) ARG190 (4.12)	
α-Humulene	− 5.8	–	PHE41 (4.65)	–
			ARG190 (5.42)	
Borneol	− 4.5	ALA100 (2.39)	ALA94 (4.85)	–
			ALA100 (4.24)	
Humulene epoxide-II	− 5.6	LYS189 (2.54)	–	–
Caryophyllene oxide	− 5.7	–	ILE78 (4.91)	–
D-Limonene	− 5.4	–	VAL43 (3.78)	–
			ILE78 (5.49)	
			VAL120 (3.73)	
			VAL167 (3.99)	
β-Pinene	− 4.2	–	ILE94 (4.62)	–
			ALA100 (4.32)	
			ALA100 (4.29)	
β-Myrcene	− 4.9	–	ALA47 (4.30)	–
			ALA47 (4.00)	
			VAL71 (4.57)	
			ILE78 (4.94)	
Fenchyl acetate	− 4.6	HIS99 (2.24)	–	–
		SER121 (2.59)		
1-Terpineol	− 5.4	HIS99 (2.24)	–	–
		SER121 (2.59)		
Iso-aromadendrene epoxide	− 6.4	–	PHE41 (4.96)	–
			ILE186 (4.87)	
			LYS189 (4.20)	
			ARG190 (4.61)	
Humulenol-II	− 6.0	–	HIS38 (4.66)	–
			PHE41 (5.39)	
			ILE86 (5.15)	

**Figure 6:**
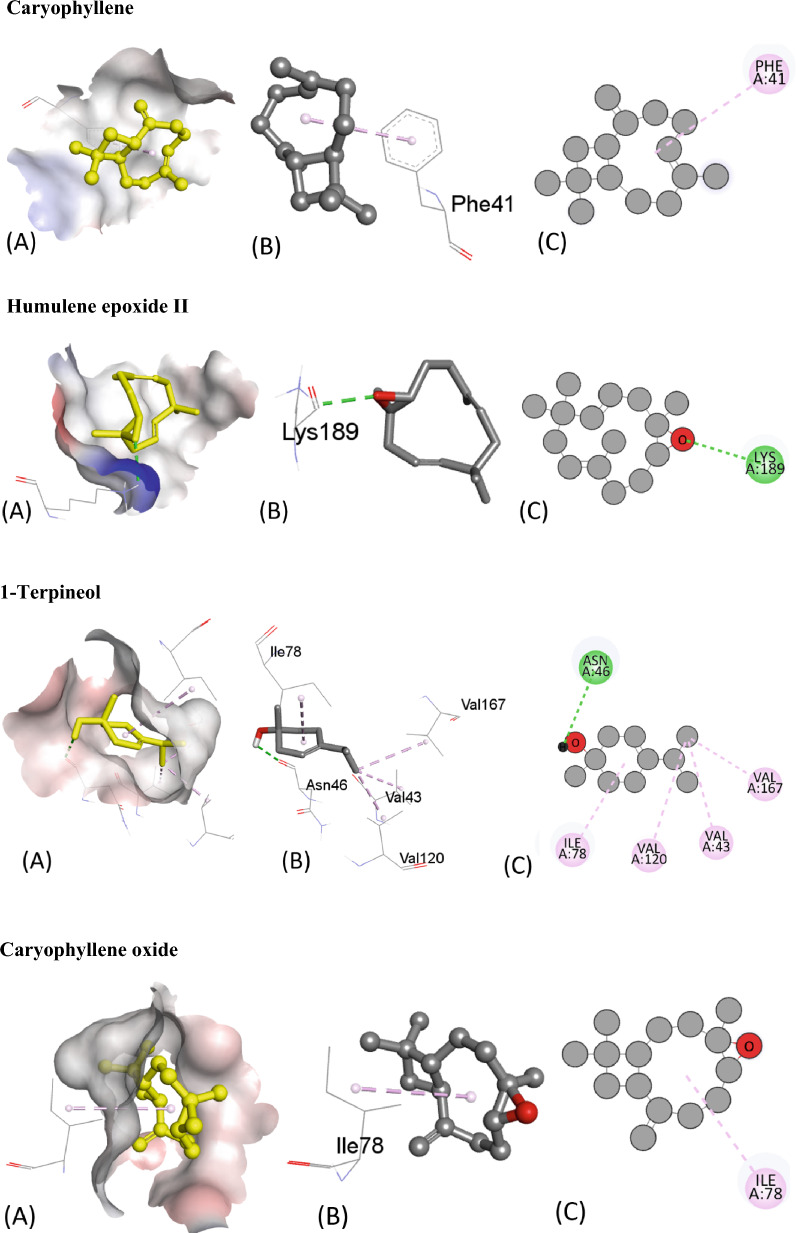

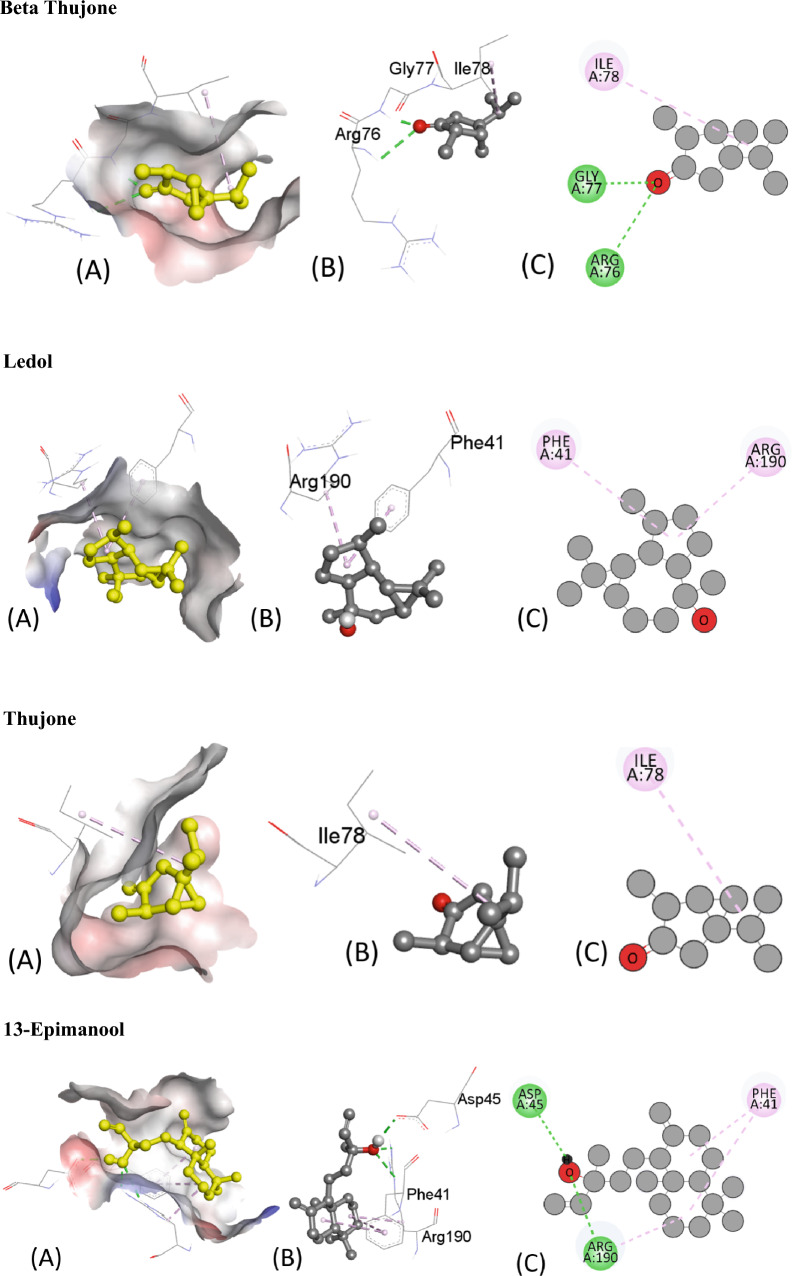

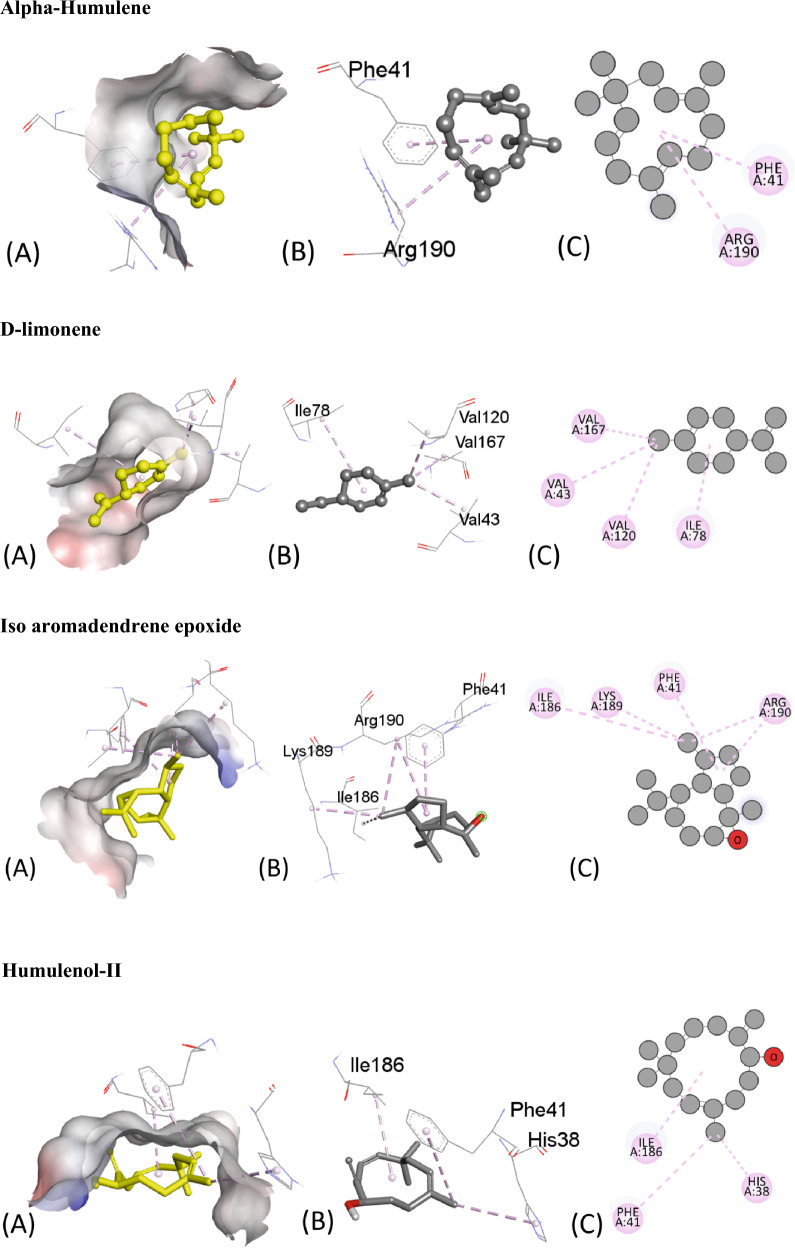
The putative 2D and 3D binding mode of investigated compounds with 1AJ6. (**A**) Binding pocket surface, (**B**) 3D binding interactions with protein, (**C**) 3D binding interactions with protein.

The residue and distance between the ligand and protein target shows the hydrogen-binding interaction. Hydrogen bonds are intermolecular forces between a hydrogen atom in the ligand and an electronegative atom in the protein. The distance between the hydrogen atom and the electronegative atom is critical, and a shorter distance indicates a stronger hydrogen bond. For instance, the ligand “13-Epimanool” forms hydrogen bonds with ASP45, ARG190, and PHE41 residues with distances of 2.18 Å, 2.98 Å, and 2.169 Å, respectively. The residue and distance between the ligand and protein target show hydrophobic interaction. Hydrophobic interactions occur when non-polar parts of the ligand and protein come in contact. A smaller distance between the non-polar parts indicates a more vital hydrophobic interaction. For example, the ligand “β-Myrcene” forms hydrophobic interactions with ALA47, VAL71, and ILE78 residues with distances of 4.30 Å, 4.00 Å, and 4.94 Å, respectively. The residue and distance between the ligand and protein target also shows the electrostatic interaction. Electrostatic interactions occur between charged atoms in the ligand and protein. A smaller distance between charged atoms indicates a stronger electrostatic interaction. For instance, the ligand “β-Thujone” forms electrostatic interactions with ARG76, GLY77, and ILE78 residues with distances of 3.05 Å, 1.90 Å, and 5.05 Å, respectively. All interactions are also shown in Fig. 6.

Similarly, Table 6 provides the results of molecular docking simulations of various ligands with an antimicrobial protein target, 1R4U. The docking score, hydrogen bonding, hydrophobic and electrostatic interactions with their respective distances in Angstrom are presented for each ligand. The binding affinity or ΔG value, which indicates the strength of interaction between the ligand and the protein, ranges from − 7.2 to − 4.6 kcal/mol. A lower ΔG value indicates a stronger binding affinity between the ligand and protein. Hydrogen bonding is an important interaction between ligands and protein residues, and this table shows the residue and the distance of the hydrogen bond formed with each ligand. Hydrophobic interaction and electrostatic interaction are also important factors that influence ligand–protein binding. This table lists the residues involved in these interactions and their respective distances.

**Table 6:** Molecular Docking score, Hydrogen binding, hydrophobic and electrostatic interactions with distances in Angstrom for investigated ligands using protein target 1R4U.

Ligands with 1R4U	Binding Affinity, ΔG (kcal/mol)	Hydrogen-Binding interaction, Residue (Distance Å)	Hydrophobic Interaction, Residue (Distance Å)	Electrostatic interaction, Residue (Distance Å)
Camphor	− 5.2	ARG176 (2.62)	–	–
		ARG176 (3.65)		
β-Thujone	− 5.2	TRP106 (2.01)	ARG105 (5.19)	–
			ARG128 (4.93)	
			TRP208 (3.83)	
Eucalyptol	− 5.1	ARG105 (2.76)	TRP208 (3.92)	–
			ARG105 (4.83)	
			ARG128 (5.01)	
α-Pinene	− 5.0	–	TRP208 (3.88)	–
			CYS103 (5.02)	
			ARG105 (4.57)	
			ARG105 (4.25)	
Ledol	− 6.7	–	PHE41 (4.96)	–
			ILE186 (4.87)	
			LYS189 (4.20)	
			ARG190 (4.66)	
Thujone	− 5.2	TRP106 (1.99)	TRP208 (3.82)	–
			ARG105 (5.17)	
			ARG128 (4.90)	
Camphene	− 4.8	–	CYS103 (4.98)	–
			ARG105 (4.88)	
			ARG105 (4.11)	
			ARG128 (4.36)	
Caryophyllene	− 6.5	–	ARG105 (5.20)	–
13-Epimanool	− 6.8	THR107 (2.06)	–	–
α-Humulene	− 4.6	–	PHE41 (4.65)	–
			ARG105 (5.42)	
Borneol	− 5.1	–	TRP208 (3.74)	–
			ARG105 (4.42)	
			TRP208 (5.49)	
Humulene epoxide-II	− 4.7	LYS186 (2.54)	–	–
Caryophyllene oxide	− 6.7	ARG105 (2.45)	ARG105 (4.75)	–
D-Limonene	− 5.0	–	CYS103 (5.49)	–
			ARG105 (3.98)	
			ARG128 (4.79)	
			ARG105 (4.79)	
			ARG128 (4.31)	
			TRP208 (4.91)	
			TRP208 (4.83)	
β-Pinene	− 5.0	–	CYS103 (4.1)	–
			TRP208 (3.72)	
			ARG105 (4.61)	
			ARG105 (4.36)	
			ARG128 (4.49)	
			TRP208 (5.34)	
β-Myrcene	− 4.6	–	TRP208 (3.77)	–
			Pro76 (5.24)	
			CYS103 (3.96)	
			CYS103 (3.73)	
			ARG128 (4.76)	
			PRO76 (4.09)	
			CYS103 (4.24)	
			ARG105 (4.54)	
			ARG128 (4.47)	
			TYR30 (5.08)	
			TYR30 (5.18)	
Fenchyl acetate	− 5.8	TRP106 (2.67)	CYS103 (5.15)	–
		THR107 (2.47)	TYR30 (5.42)	
1-Terpineol	− 5.4	–	CYS103 (5.47)	–
			TYR30 (4.81)	
Iso-aromadendrene epoxide	− 7.2	TRP106(1.82)	CYS103 (4.12)	–
			PRO76 (4.17)	
			CYS103 (3.94)	
			TYR30 (4.98)	
			TYR30 (5.46)	
Humulenol-II	− 7.0	TRP106 (2.62)	TRP208 (3.57)	–
		HIS104 (3.61)	PRO76 (4.82)	
			CYS103 (3.94)	
			TYR30 (5.08)	

**Figure 7:**
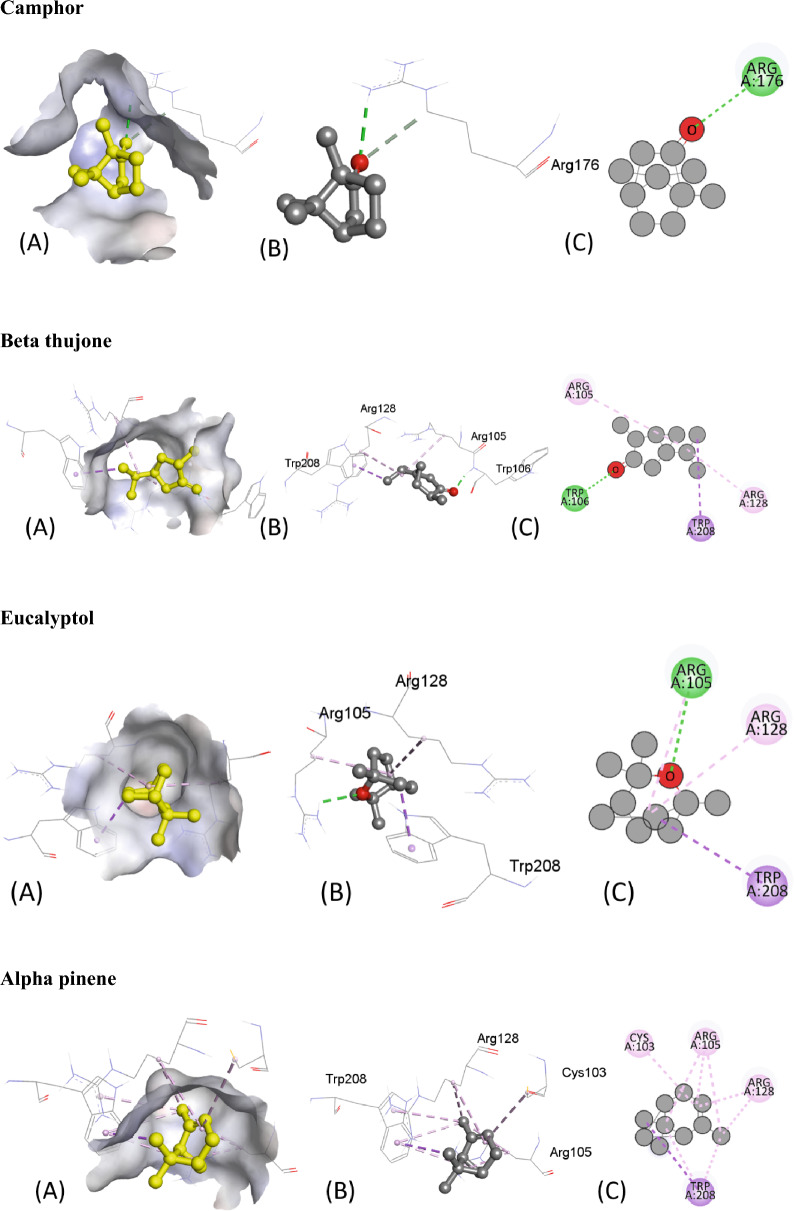

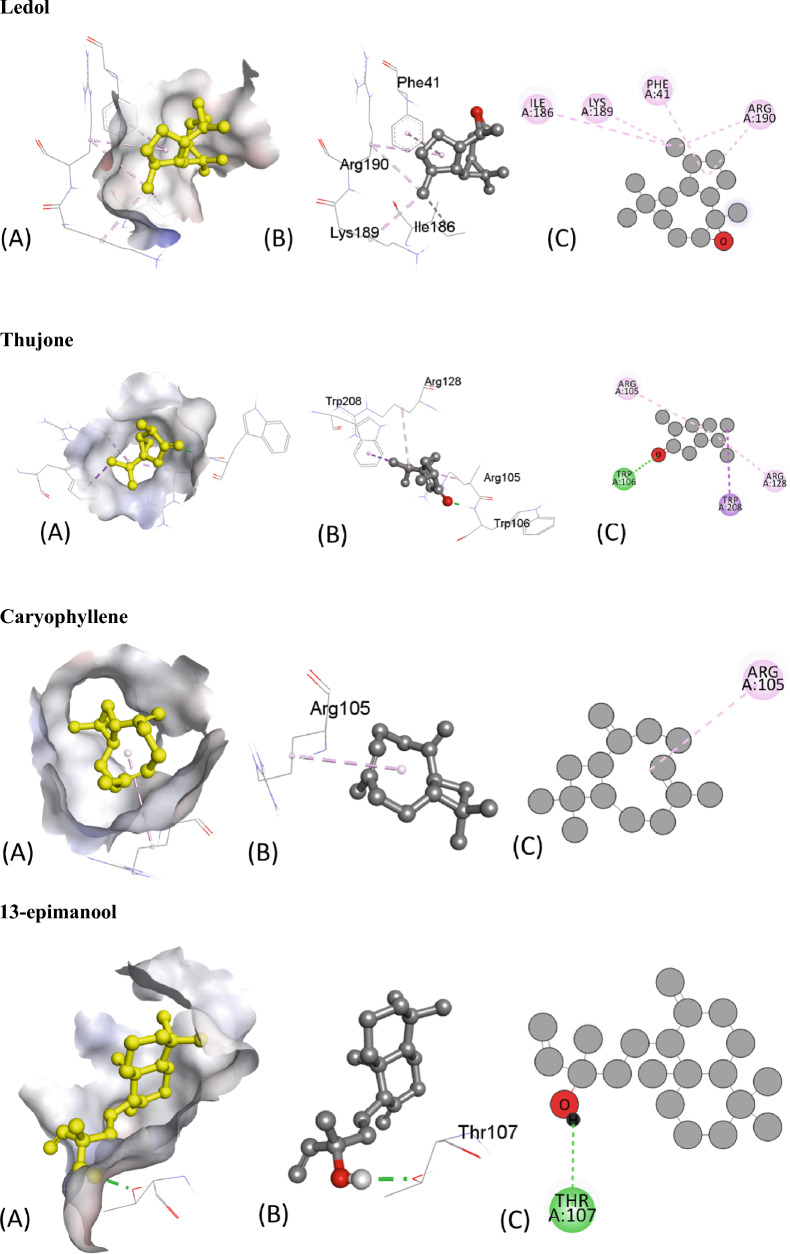

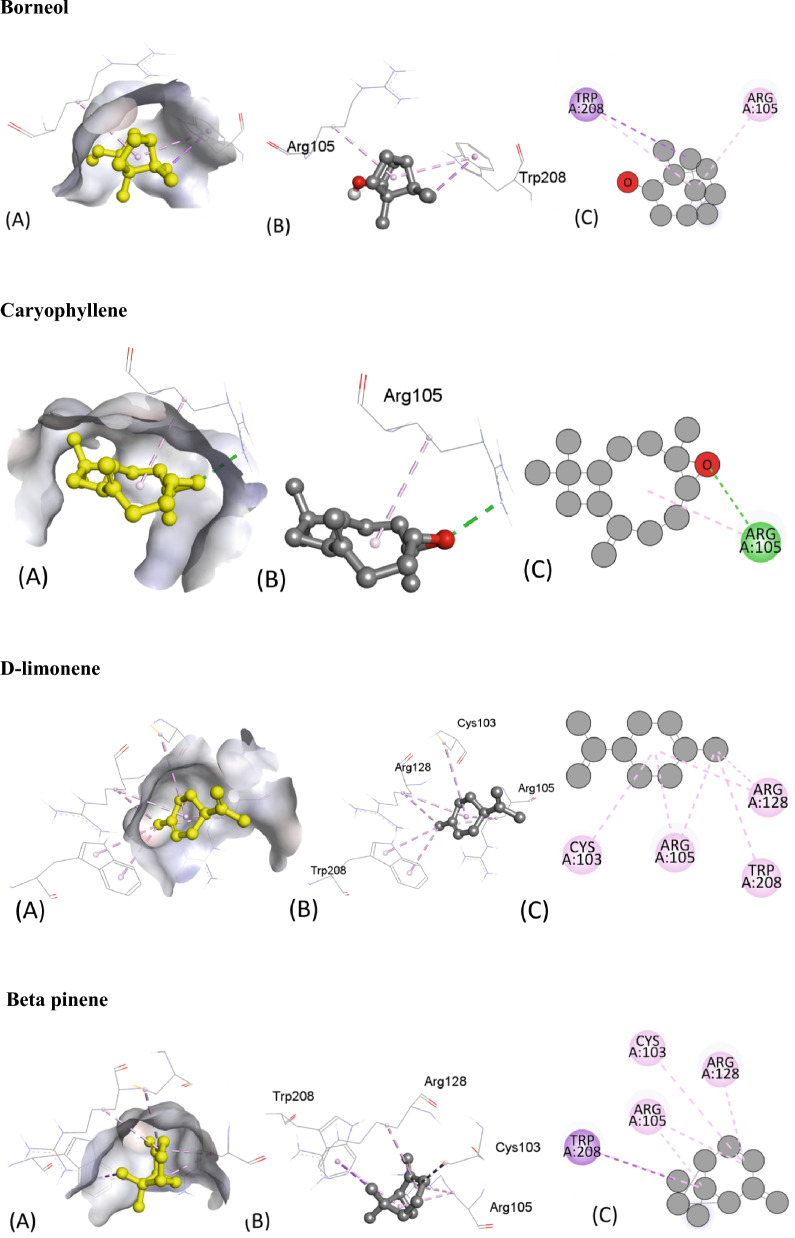

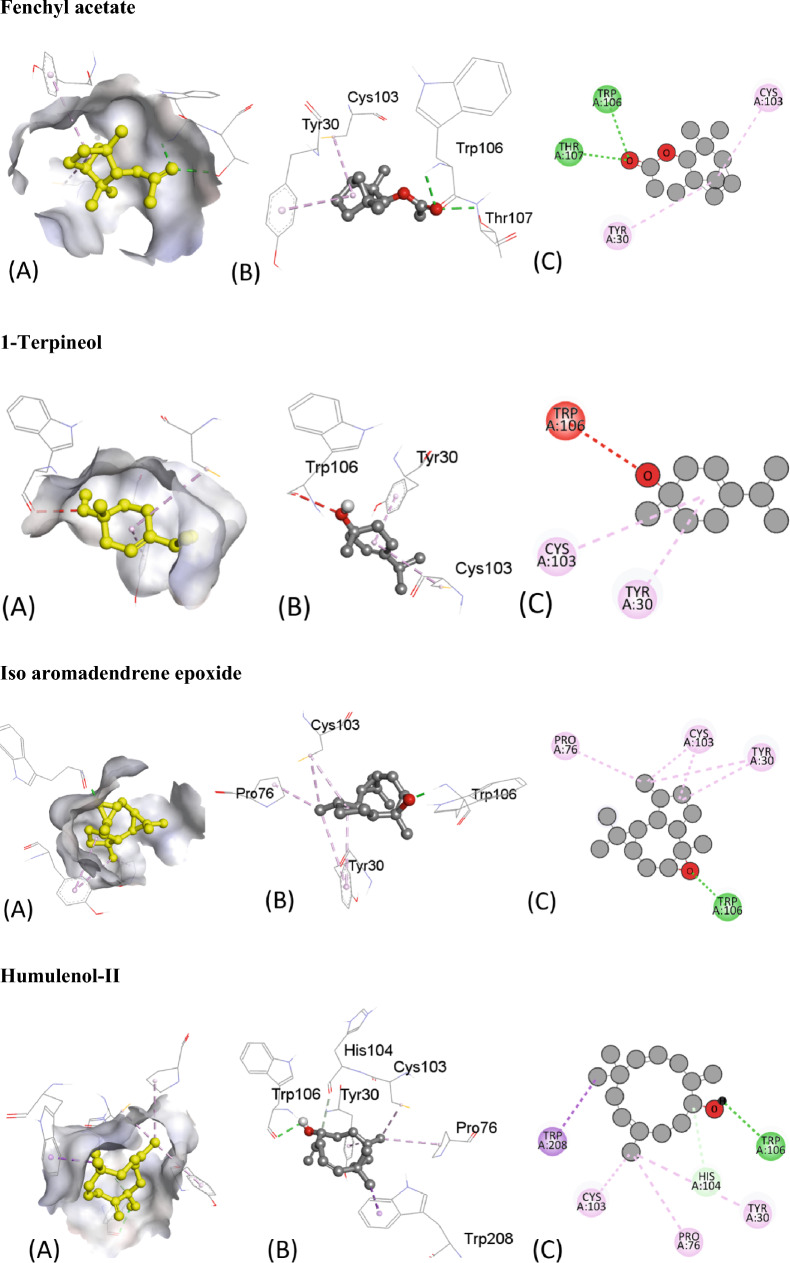
The putative 2D and 3D binding mode of investigated compounds with 1R4U. (**A**) Binding pocket surface, (**B**) 3D binding interactions with protein, (**C**) 3D binding interactions with protein.

The ligands can be compared based on their binding affinities and the types of interactions formed with the protein residues. Ligands such as Humulenol-II and Iso-aromadendrene epoxide show the strongest binding affinities with ΔG values of − 7.0 and − 7.2 kcal/mol, respectively. Both ligands also form hydrogen bonds with the protein residues TRP106 and HIS104, respectively. In contrast, ligands such as β-Myrcene and α-Humulene have weaker binding affinities with ΔG values of − 4.6 kcal/mol and − 4.8 kcal/mol, respectively. β-Myrcene forms multiple hydrophobic interactions with various protein residues, while α-Humulene forms only one hydrophobic interaction with PHE41. Figure 7 represents all the ligands in 2D and 3D interactive ways for clarification.

In comparison, the molecular docking results in Tables 5 and 6 suggest that some of the tested ligands have strong binding affinities and favorable interactions with the antioxidant and antimicrobial protein targets. These findings provide valuable insights for developing new compounds with potential antioxidant and antimicrobial activity. The ligand “Iso-aromadendrene epoxide” was found to have the strongest binding affinity with the antioxidant protein target 1AJ6. In contrast, the ligands “Humulenol-II” and “Iso-aromadendrene epoxide” showed the most potent binding affinities with the antimicrobial protein target 1R4U. Interestingly, “Iso-aromadendrene epoxide” was found to have a strong binding affinity with both protein targets, indicating its potential as a multi-targeted drug candidate.

Furthermore, the analysis of the interactions between the ligands and protein residues provides insights into the specific mechanisms of ligand–protein binding. These findings can be used to guide the rational design and optimization of new compounds with improved antioxidant and antimicrobial activity. Overall, the results of these molecular docking simulations are promising and provide a starting point for further experimental validation of the identified ligands.

## Conclusions

This study was carried out to determine the phytochemical composition, antioxidant activity, and antimicrobial effect of essential oils of *S. lavandulifolia* subsp. *mesatlantica* and *S. officinalis* L. Our results showed 10 and 20 chemicals in essential oils of *S. officinalis* and *S lavandulifolia*, respectively, which are diverse in terms of types but fewer when compared with previous studies. The recorded constituents were significantly higher in *S. officinalis* compared to *S. lavandulifolia*. These chemical compounds support an interesting antioxidant capacity in analyzed essential oils of both *Salvia* species. The Scavenging of the Free Radical DPPH was significantly superior in *S. officinalis*, while Total Antioxidant Capacity (TAC) was higher in *S. lavandulifolia*.

On the other hand, essential oils of both *Salvia* species showed interesting antimicrobial activity against bacteria and yeast. The highest inhibition rates were recorded for *S. officinalis* compared to *S. lavandulifolia*. The results provide relevant evidence on the phytochemical composition of *S. officinalis* and *S. lavandulifolia* in Morocco, the first for medicinal plants data in this North African country. Equally, the recorded antimicrobial activity is the first for *S. lavandulifolia* worldwide. Therefore, this study will benefit other comparative studies on the same subject. However, additional research is required to evaluate the phytochemical components of native and commercialized variants of these plants. Suppose their compositions would be similar (native and cultivated). In that case, they will promote the extensive use of cultivated materials and the protection of wild species, which is suggested to be beneficial for protecting wild florae. Information on Ligand–protein interactions for antioxidant and antimicrobial proteins provided valuable details on binding affinities and interactions produced that can be highly useful as an initial step of drug discovery. Creating new treatments can benefit from examining binding affinities and the many kinds of interactions produced.

## Data Availability

All data generated or analyzed during this study are included in this published article.
